# Purinergic Signaling in Neuroinflammation

**DOI:** 10.3390/ijms222312895

**Published:** 2021-11-29

**Authors:** Dmitry Aminin, Peter Illes

**Affiliations:** 1 G.B. Elyakov Pacific Institute of Bioorganic Chemistry, Far Eastern Branch of the Russian Academy of Sciences, 690022 Vladivostok, Russia; daminin@piboc.dvo.ru; 2 Department of Biomedical Science and Environmental Biology, Kaohsiung Medical University, Kaohsiung City 80708, Taiwan; 3 Rudolf Boehm Institute for Pharmacology and Toxicology, University of Leipzig, 04107 Leipzig, Germany; 4 International Collaborative Center on Big Science Plan for Purine Signaling, Chengdu University of TCM, Chengdu 610075, China

ATP is stored in millimolar concentrations within the intracellular medium but may be released to extracellular sites either through the damaged plasma membrane or by means of various transporters. Extracellular ATP or its enzymatic breakdown products, ADP, AMP, and adenosine, may then stimulate a range of membrane receptors (Rs). These receptors are classified as belonging to two types termed P2 or P1. P2Rs can be, in addition, subdivided into the ligand-activated P2X and the G protein-coupled P2Y types. Adenosine acts on the P1 type of receptor. A further classification identifies seven mammalian subtypes of P2X1-7 and eight mammalian subtypes of P2YRs (P2Y1, P2Y2, P2Y4, P2Y6, P2Y11, P2Y12, P2Y13, P2Y14). P1Rs are either positively (A2A, A2B) or negatively (A1, A3) coupled to adenylate cyclase via the respective G proteins. Already, such a high number of receptors suggests that purine-mediated effects at the cellular but especially whole organism level have an immense variability. Whereas P2XRs respond only the ATP, P2YRs are sensitive to ATP/ADP, UTP/UDP, or UDP–glucose. Inspection of some articles in this Special Issue will teach us that the nucleoside guanosine probably possesses a receptor of its own, that nucleotides can be gradually degraded metabolically to functionally active nucleotides/nucleosides (see above), and indirect effects by stimulating the synthesis or decomposition of purines/pyrimidines may also increase functional diversity. Eventually, P2/P1Rs may interact both with each other as well as with other neurotransmitter receptors. It is, of course, important to note that, in many cases, receptor (sub)type-preferential agonists and highly selective antagonists are available for pharmacological analysis.

The fascinating complexity of the “purinome” (the cluster of agonists, receptors, and enzymes participating in purinergic signaling) and the involvement of its components in (patho)physiological functions underline their regulatory importance. Defective P2XRs are causative factors, e.g., in male infertility (P2X1), hearing loss (P2X2), pain/cough (P2X3), neuropathic pain (P2X4), inflammatory bone loss (P2X5), and faulty immune reactions (P2X7). Purinergic signaling may regulate immune reactions through P2/P1Rs situated at immune cells in the periphery (macrophages, lymphocytes) and in the central nervous system (microglia). P2X4,7 and P2Y4,6,12, as well as A2A,3Rs, are located at microglia in the CNS. They steer microglial process motility, microglial migratory activity, microglial phagocytosis (pinocytosis), and the release of pro-inflammatory cytokines, chemokines, growth factors, proteases, reactive oxygen and nitrogen species, cannabinoids, and probably also the secretion of excitotoxic ATP and glutamate. Consequently, the “purinome” is involved in the neurodegenerative illnesses Alzheimer’s disease, Parkinson’s disease, Huntington’s disease, multiple sclerosis, amyotrophic lateral sclerosis, neuropathic pain, post-epilepsy and post-ischemia neuronal damage, etc. These latter findings prompted the two editors of this Special Issue to invite a few experts to contribute their ideas on purinergically induced neuroinflammation. This choice of publications consists of two original articles and eight reviews, providing new insights to our present knowledge. 

One of the original papers by Braune et al. [1] deals with the G protein-coupled GPR17, which was initially discovered as an orphan receptor and was found to be a target of both cysteinyl-leukotrienes and the uracil nucleotides uridine, UDP, and UDP–glucose. Montelucast, a selective antagonist of GPR17, largely facilitated the outgrowth of neuronal fiber networks from the substantia nigra/ventral tegmentum to the prefrontal cortex in an organotypic co-culture system. This effect appeared to be due to antagonism of endogenous ligands activating GPR17, because a selective agonist of this receptor, PSB-16484, reversed growth promotion by the GPR17 antagonist Montelucast. Another original article of Sophocleous et al. [2] proved the presence of P2Y2 and P2X4Rs at a canine macrophage cell line. P2Y2Rs mediated the mobilization of Ca^2+^ from its intracellular stores, while the low levels of P2X4Rs might modulate this effect. Canine cells are feasible alternatives to rodent cell systems for drug approval procedures.

The residual review articles deal with ischemia, neonatal seizures, chronic pain, retinal disorders, multiple sclerosis, and osteogenesis/adipogenesis. Coppi et al. [3] report on neuronal damage generated by cerebral ischemia (occlusion of the middle cerebral artery) in the whole animal or in vitro by oxygen–glucose deprivation in hippocampal slices. A2BRs were found to participate in the early glutamate-mediated excitotoxicity responsible for neuronal and synaptic loss in the CA1 hippocampal cells. By contrast, after the ischemic stimulus, the same receptors have protective roles in tissue damage and functional impairment. In this context, Chojnowski et al. [4] referred to the fact that guanosine, which is released under brain ischemia or trauma into the extracellular milieu, counteracted the destructive events occurring during ischemic conditions (e.g., glutamatergic excitotoxicity, reactive oxygen and nitrogen species production). Neonatal seizures are a particularly drug-resistant form of epilepsy. Menéndez Méndez et al. [5] provided data suggesting that P2X7R antagonists, previously investigated in adult epilepsy, have the most promise in neonatal seizures.

Vincenzi et al. [6] contributed extensively to the role of adenosine in pain regulation. Most of the anti-nociceptive effects of adenosine have been found to depend upon A1Rs located at peripheral, spinal, and supra-spinal sites. A2A and A2BRs have been found to be more controversial, since their activation led to both pro- and anti-nociceptive consequences. More recently, allosteric activators have been proposed to improve efficacy and limit side effects of endogenous adenosine. Trapero et al. [7] have addressed endometriosis-associated pain, which depended on inflamed endometrial tissue localized outside the uterine cavity. Altered extracellular ATP hydrolysis, due to changes in ectonucleotidase activity, leads to accumulation of ATP in the endometriosis microenvironment and activates pain-inducing P2X3Rs at sensory neurons. Pharmacological inhibition of this receptor-type appears to be an adequate therapeutic option. 

Sidoryk-Wegrzynowicz and Struzyska [8] have dedicated their review to astroglial and microglial purinergic P2X7Rs as major contributors to neuroinflammation in multiple sclerosis. Hársing et al. [9] discussed findings demonstrating that energy deprivation causes in the retina an increased release of the excitotoxic ATP and glutamate, mediated by P2X7 and NMDARs, respectively. P2YR agonists facilitate the uptake of glycine by the glycine transporter 1; the resulting lower extracellular concentrations of the NMDAR co-agonistic glycine reduces neurodegenerative events in the retina. Finally, Eisenstein et al. [10] analyze the dichotomous effects of the two G protein-coupled stimulatory and the two G protein-coupled inhibitory adenosine receptors on adipogenesis and osteogenesis within the bone marrow to maintain bone health, as well as its relationship to obesity. 

We are confident that, already, this limited number of papers will emphasize the great importance of purinergic signaling in neuroinflammation and the requirement for therapies directed to the involved receptors, release mechanisms, and metabolizing enzymes. It is our strong conviction that the subject deserves a subsequent Special Issue with the same or even higher number of contributions. For this issue, we intend to invite further specialists to elucidate research fields complementary to the presently discussed ones.

## Data Availability

The data presented in this study are available upon request from the corresponding authors of the respective publications.
